# Creating an Educational Neuropsychiatry Network

**DOI:** 10.1192/bjo.2026.11317

**Published:** 2026-06-30

**Authors:** Rachel Gorny, Mohammad Zia Ul Haq Katshu, Alina Alina Voinea

**Affiliations:** 1 Derbyshire Healthcare NHS Foundation Trust, Derby, United Kingdom; 2 University of Nottingham, Nottingham, United Kingdom; 3 Notts Healthcare NHS Foundation Trust, Nottingham, United Kingdom

## Abstract

**Aims:**

Tobuild a Multiprofessional Neuropsychiatry educational network. To provide high standard educational meetings through complex case presentations which promote neuropsychiatry across the East Midlands.

**Methods:**

Neuropsychiatry as a sub-speciality is not endorsed by the GMC, and as such formal training pathways do not exist for psychiatrists in the UK. The Neuropsychiatry Discussion Group (NPDG thereafter) is a monthly forum designed for clinicians across all levels and specialties to explore complex neuropsychiatric presentations. Each session a clinician presents a complex case that challenges diagnostic frameworks and treatment strategies. In the past 12 months we have expanded the network, hosted guest speakers from experts to trainees with an interest in neuropsychiatry and formalised the programme. We regularly have 70+ attendees across neighbouring trusts in the East Midlands and are continuously expanding the network to foster professional collaboration.

We base the programme on the RCPsych Faculty of Neuropsychiatry suggested syllabus to ensure curriculum coverage over the year and have developed a standardised feedback form for the sessions using a Likert scale as well as free text to collect data.

**Results:**

Analysis of participant feedback across multiple sessions demonstrated strongengagement and consistently high satisfaction with the educational content and delivery.The programme successfully incorporated a broad range of presenters spanning training grades and seniority, from CT1 trainees to Professors of Neuropsychiatry, reflecting an inclusive academic culture and exposure to varied perspectives. This breadth was frequently reflected in comments.

Quantitative data showed that participants rated the clarity of session objectives, clinical and academic relevance, and effectiveness of teaching methods predominantly at levels 4 and 5, indicating a high degree of perceived educational value and quality.

Qualitative feedback reinforced these findings. Attendees frequently emphasised the strengths of the case-based format, praising the sessions for their real-world clinical applicability, multidisciplinary input, and opportunities for collaborative discussion. The format was noted to support deep, reflective learning, particularly in areas where diagnostic and treatment paradigms are complex or evolving.

**Conclusion:**

Ongoing evaluation will remain integral to the development of the group. We will continue to systematically collect and analyse participant feedback across sessions.

We are working with neighbouring trusts and organisations in order that attendance at the sessions can be used for CPD activity.

We aim to continue to widen the NPDG network, therefore fostering rich, cross-specialty dialogue. We wish to deepen clinical insight, share perspectives, and enhance collaborative care across the region.

